# Characterization of optogenetically activated inhibitory inputs onto cholinergic motor neurons in the spinal dorsolateral nucleus

**DOI:** 10.14814/phy2.70703

**Published:** 2025-12-19

**Authors:** Tadanobu C. Kamijo, Sergei Karnup, Kanako Matsuoka, Shingo Kimura, Minoru Miyazato, Stephanie L. Daugherty, Jonathan M. Beckel, Naoki Yoshimura

**Affiliations:** ^1^ Department of Urology University of Pittsburgh School of Medicine Pittsburgh Pennsylvania USA; ^2^ Department of Systems Physiology, Graduate School of Medicine University of the Ryukyus Okinawa Japan; ^3^ Department of Pharmacology and Chemical Biology University of Pittsburgh School of Medicine Pittsburgh Pennsylvania USA

**Keywords:** external urethral sphincter, inhibitory synaptic transmission, optogenetics, patch‐clamp electrophysiology, spinal motor neurons

## Abstract

Inhibitory control of external urethral sphincter motor neurons (EUS‐MNs) in the spinal dorsolateral nucleus (DLN), which corresponds to a portion of Onuf's nucleus in humans, is essential for normal micturition by inducing EUS relaxation during voiding; yet synaptic mechanisms remain poorly characterized. Using neonatal mice P8‐P12, we developed a slicing technique—cutting spinal cords at 150° from the coronal plane (30° from the horizontal plane in the agarose block), for maximizing EUS‐MNs captured per slice. Using transgenic mice co‐expressing channelrhodopsin‐2 in inhibitory interneurons (VGAT‐ChR2) and GFP in cholinergic neurons (ChAT‐GFP), we investigated inhibitory synaptic transmission onto EUS‐MNs. Optogenetic activation evoked robust inhibitory postsynaptic potentials (IPSPs), classified as sustained or transient based on temporal profiles. Pharmacology revealed that sustained IPSPs contained both glycinergic and GABAergic components, while GABAA receptors predominantly mediated transient IPSPs. Strychnine (1 μM) selectively blocked glycinergic transmission, while bicuculline (10 μM) eliminated GABAergic components. Insensitivity to glutamatergic antagonists (CNQX and AP5) confirmed purely inhibitory responses. Our findings demonstrate segregation of inhibitory inputs onto EUS‐MNs, with glycinergic and GABAergic transmission contributing to sustained and transient inhibition, respectively, establishing the methodological foundation for investigating inhibitory circuit dynamics in pathological conditions such as spinal cord injury with deficient inhibitory control.

## 1 INTRODUCTION

The coordinated control of the lower urinary tract (LUT) is essential for normal micturition and continence. This coordination depends on precise neural mechanisms that regulate the reciprocal activity of the urinary bladder and the external urethral sphincter (EUS). The successful storage and release of urine rely on the synchronized contraction of the bladder (detrusor muscle) and relaxation of the EUS during voiding, or conversely, relaxation of the bladder and contraction of the EUS during storage (de Groat et al., 2015). This coordination is orchestrated by neural circuits in the brain and the spinal cord, with the latter playing a crucial role in integrating supraspinal commands with peripheral sensory feedback.

Spinal motor neurons innervating the EUS are known to be located in Onuf's nucleus in the spinal ventral horn. While motor neurons of the EUS (EUS‐MNs) and the external anal sphincter (EAS‐MNs) are located close to each other within Onuf's nucleus in various species including cats, chimpanzees, and humans (Karnup et al., 2024; Schellino et al., 2020), Onuf's nucleus in rats and mice is split into two different clusters: namely, dorsolateral nucleus (DLN) and dorsomedial nucleus (DMN) in the L6‐S1 spinal cord, which contains EUS‐MNs and EAS‐MNs, respectively (Karnup et al., 2024; McKenna & Nadelhaft, 1986). Furthermore, EUS‐MNs in the DLN receive multiple synaptic inputs, both excitatory and inhibitory, which regulate their activity during the micturition cycle. Inhibitory inputs to EUS‐MNs are particularly critical for facilitating EUS relaxation during voiding, enabling efficient bladder emptying. Without this inhibition, conditions such as detrusor‐sphincter dyssynergia can occur, where the sphincter fails to relax appropriately during bladder contractions, resulting in impaired voiding (Karnup & de Groat, 2020; Shefchyk et al., 1998).

The inhibitory control of EUS‐MNs is primarily mediated by glycinergic and GABAergic neurotransmission. Glycine receptors are abundantly expressed on EUS‐MNs (Becker et al., 1994), and glycinergic inhibition is essential for suppressing EUS activity during micturition. The importance of glycinergic inhibition in normal micturition has been demonstrated through pharmacological studies where strychnine, a glycine receptor antagonist, disrupts EUS relaxation and impairs voiding (Shefchyk et al., 1998). Similarly, GABA, acting via GABA_A_ receptors, contributes to the inhibitory control of EUS activity, and hypofunction of GABAergic mechanisms can lead to voiding dysfunctions, particularly after spinal cord injury (SCI) (Miyazato et al., 2009). Understanding these inhibitory mechanisms is particularly relevant for pathological conditions such as spinal cord injury, where deficient inhibitory control contributes to detrusor‐sphincter dyssynergia (Hashimoto et al., 2024; Hsiang & Vizzard, 2024). Additionally, while inhibitory transmission undergoes developmental maturation, studying these mechanisms in neonatal tissue (P8‐12) provides methodological advantages for establishing the experimental approach, as discussed in our previous work (Karnup et al., 2024).

Recent advances in optical methods, particularly optogenetics, have revolutionized the study of inhibitory synaptic transmission in the spinal cord. Optogenetic approaches allow precise control of specific neuronal populations with millisecond temporal resolution, enabling researchers to dissect the complex neural circuits involved in EUS control (Montgomery et al., 2016). Transgenic mouse models expressing channelrhodopsin‐2 (ChR2) under the control of specific promoters, such as the vesicular GABA transporter (VGAT‐ChR2 mice; Stock No. 014548, Jackson Laboratory, Bar Harbor, ME, USA) or choline acetyltransferase (ChAT‐GFP mice; Stock No. 007902, Jackson Laboratory), which do not express ChR2, have been particularly valuable in these investigations (Zhao et al., 2011). VGAT‐ChR2 mice express ChR2 in GABAergic and glycinergic neurons, allowing for their selective activation with blue light stimulation. This enables precise control of inhibitory neurons and facilitates studying their connections and functional roles in spinal motor circuits. Similarly, ChAT‐GFP mice, which express green fluorescent protein in cholinergic neurons, allow for the visualization and targeted recording from motor neurons and other cholinergic cells in the spinal cord (Tallini et al., 2006).

These transgenic models offer several advantages over traditional electrophysiological methods for studying inhibitory synaptic transmission. The specificity of optogenetic activation reduces the complexity of interpreting mixed signals from electrical stimulation, which often activates multiple neuronal populations simultaneously. Additionally, controlling neuronal activity with light allows for more precise temporal manipulation of inhibitory inputs, facilitating the study of their dynamic interactions with other synaptic inputs and their role in motor output patterns (Tseng et al., 2015).

In this study, we employed optogenetic approaches using VGAT‐ChR2 and ChAT‐GFP mouse models to investigate the properties of inhibitory synaptic inputs to EUS‐MNs in the DLN, which is a portion of Onuf's nucleus in humans (Karnup et al., 2024). We specifically focused on characterizing glycinergic and GABAergic components of these inputs. By combining optogenetic stimulation with patch‐clamp recording, we aim to provide detailed insights into the inhibitory control of EUS‐MNs, which is crucial for understanding the EUS‐controlling mechanism underlying normal LUT function and the pathophysiology of LUT dysfunction.

## 2 MATERIALS AND METHODS

### 2.1 Animals

Transgenic mice expressing ChAT–GFP in cholinergic neurons (Stock No. 007902, Jackson Laboratory) and VGAT–ChR2 in inhibitory neurons were obtained by cross‐breeding these two strains (Stock No. 014548, Jackson Laboratory), so that some offspring expressed GFP in cholinergic neurons and ChR2 in inhibitory neurons. Both male and female pups were included. Animals were bred in‐house at the University of Pittsburgh, with the day of birth designated as postnatal Day 0 (P0). Experiments were conducted on postnatal Days 8–12 (P8–P12).

Mice were housed in individually ventilated cages (IVCs) under a 12–h light/dark cycle (lights on at 7:00 a.m., off at 7:00 p.m.) at 22 ± 1°C and 50 ± 10% humidity. Food (ProLab Iso Pro RMH 3000, cat. no. 5P76, LabDiet, St. Louis, MO, USA) and water were available ad libitum. Breeding was conducted using harem mating (ChAT‐GFP homozygous male with one or two VGAT‐YFP‐ChR2 homo‐ or heterozygous females), and pregnant females were separated into individual cages prior to parturition to avoid overcrowding. Thus, some cross‐breeding offspring expressed both ChAT‐GFP and VGAT‐YFP‐ChR2 in cholinergic and GABA/glycinergic neurons, respectively. All procedures were approved by the Institutional Animal Care and Use Committee (IACUC) of the University of Pittsburgh (Protocol No. 22061313, Modification IML–22061313–2, PHS Assurance D16–00118) and complied with the ARRIVE and NIH guidelines.

### 2.2 Slice preparation

**FIGURE 1:**
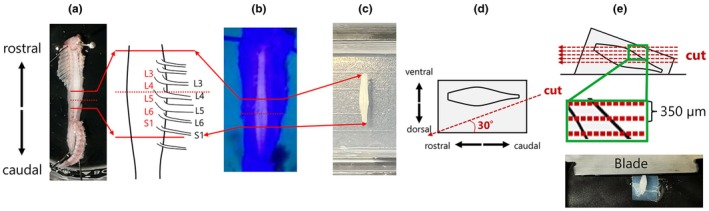
Schematic presentation of preparations of oblique spinal cord slices containing EUS motor neurons. (a) After decapitation, the vertebral column is exposed and the pedicles are cut to allow removal of the spinous processes. (b) Blue light illumination reveals ChAT–GFP fluorescence in motor neurons, guiding precise trimming of the lumbosacral spinal cord. (c) The isolated spinal cord is embedded in low–melting–point agarose for stabilization. (d) The agarose block is trimmed at a 30° oblique angle from the horizontal surface to preserve dorsolateral motor columns, including EUS motor neurons in the DLN. (e) The block is sectioned into 350 μm‐thick slices using a vibratome.

Mice were deeply anesthetized with 3% isoflurane (cat. no. NDC 66794‐017‐25, Piramal Pharma Limited, Bethlehem, PA, USA) and decapitated. The spinal cord was removed and embedded in 3% low–melting–point agarose (cat. no. A20070, Research Products International Corp, Mt. Prospect, IL, USA). Oblique slices (350 μm thickness) were cut at a 30° angle from the horizontal plane using a vibratome (VT–1000, Leica Microsystems, Wetzlar, Germany) to preserve the cytoarchitecture of the DLN in the L6‐S1 spinal cord and retain EUS–MNs. This oblique slicing strategy was designed to maximize the number of motor neurons captured within a single slice, as conventional horizontal sectioning would yield significantly fewer motor neurons per slice. This approach is critical for studying the synaptic organization of the sparsely distributed motor neurons in the spinal DLN. An overview of the slicing angle and anatomical landmarks is shown in Figure 1.

Tissue slicing was performed in ice–cold, high–sucrose cutting solution bubbled with 95% O_2_/5% CO_2_. The solution composition (in mM) was: 234 sucrose (cat. no. S9378, Sigma‐Aldrich, St. Louis, MO, USA), 2.5 KCl (cat. no. P217‐500, Thermo Fisher Scientific, Waltham, MA, USA), 1.25 NaH_2_PO_4_ (cat. no. SX0710‐1, Merck KGaA, Darmstadt, Germany), 10 MgSO_4_ (cat. no. M2670, Sigma‐Aldrich), 0.5 CaCl_2_ (cat. no. CX0130‐1, EMD Science, Merck KGaA), 11 glucose (cat. no. G7021, Sigma‐Aldrich), 26 NaHCO_3_ (cat. no. MFCD00003528, Thermo Fisher Scientific) (pH 7.4). After slicing, tissues were incubated in artificial cerebrospinal fluid (ACSF) at 31 ± 0.5°C for 1 h and then stored at room temperature for at least 2 h before recording to ensure complete recovery from slicing trauma. ACSF contained (in mM): 126 NaCl (cat. no. BDH9286‐2.5KG, VWR International, Radnor, PA, USA), 2.5 KCl (cat. no. P217‐500, Thermo Fisher Scientific), 1.25 NaH_2_PO_4_ (cat. no. SX0710‐1, Merck KGaA), 1.3 MgCl_2_ (cat. no. M2670, Sigma‐Aldrich), 2.4 CaCl_2_ (cat. no. CX0130‐1, EMD Science, Merck KGaA), 10 glucose (cat. no. G7021, Sigma‐Aldrich), 26 NaHCO_3_ (cat. no. MFCD00003528, Thermo Fisher Scientific) (pH 7.4).

### 2.3 Electrophysiology

Whole–cell current–clamp recordings were made at room temperature (21°C–23°C) using a MultiClamp 700B amplifier (Molecular Devices, San Jose, CA, USA), digitized via Power1401 mk II (CED, Cambridge, UK), and controlled using Signal software (v5.11, CED). Data were sampled at 20 kHz and low pass filtered at 3 kHz.

Recordings targeted ChAT–GFP+ EUS–MNs in the DLN of the L6‐S1 spinal cord. Cells were identified using fluorescence microscopy (BX51WI, Olympus, Tokyo, Japan) equipped with a water‐immersion objective (LUMPlanFL 40×/0.80 W, Olympus) and GFP filters (excitation 460–490 nm, dichroic mirror 505 nm, emission 510–550 nm; U‐MF2, Olympus). Images were acquired using MetaMorph TL software (version 7.7.6.0, Molecular Devices, San Jose, CA, USA), and cells were patched under infrared differential interference contrast (IR–DIC) guidance.

Recording electrodes (9–12 MΩ) were pulled from borosilicate glass capillaries (1.5 mm OD, 0.86 mm ID; cat. no. BF150‐86‐10, Sutter Instrument, Novato, CA, USA) and filled with intracellular solution (in mM): 135 K‐gluconate (cat. no. G4500, Sigma‐Aldrich), 5 KCl (cat. no. P217‐500, Thermo Fisher Scientific), 10 HEPES (cat. no. H‐7637, Sigma‐Aldrich), 4 MgATP (cat. no. A9187, Sigma‐Aldrich), 0.3 NaGTP (cat. no. G8877, Sigma‐Aldrich), 0.2 EGTA (cat. no. E3889, Sigma‐Aldrich), 10 phosphocreatine (cat. no. P4635, Sigma‐Aldrich) (pH 7.2 with KOH (cat. no. P‐1767, Sigma‐Aldrich), 275 mOsm/kg).

The theoretical chloride reversal potential (ECl) was calculated using the Nernst equation: ECl = (RT/zF) × ln([Cl^−^]out/[Cl^−^]in), where R is the gas constant (8.314 J·mol^−1^·K^−1^), T is absolute temperature (295 K at 22°C), z is the valence of chloride (−1), and F is Faraday's constant (96,485 C·mol^−1^). With [Cl^−^]in = 5 mM and [Cl^−^]out = 135.9 mM, ECl was approximately −84 mV at room temperature. The liquid junction potential for the K‐gluconate‐based internal solution was estimated to be approximately 10–15 mV, consistent with values reported in the literature for similar ionic compositions (Barry, 1994; Neher, 1992).

### 2.4 Optogenetic stimulation

**FIGURE 2:**
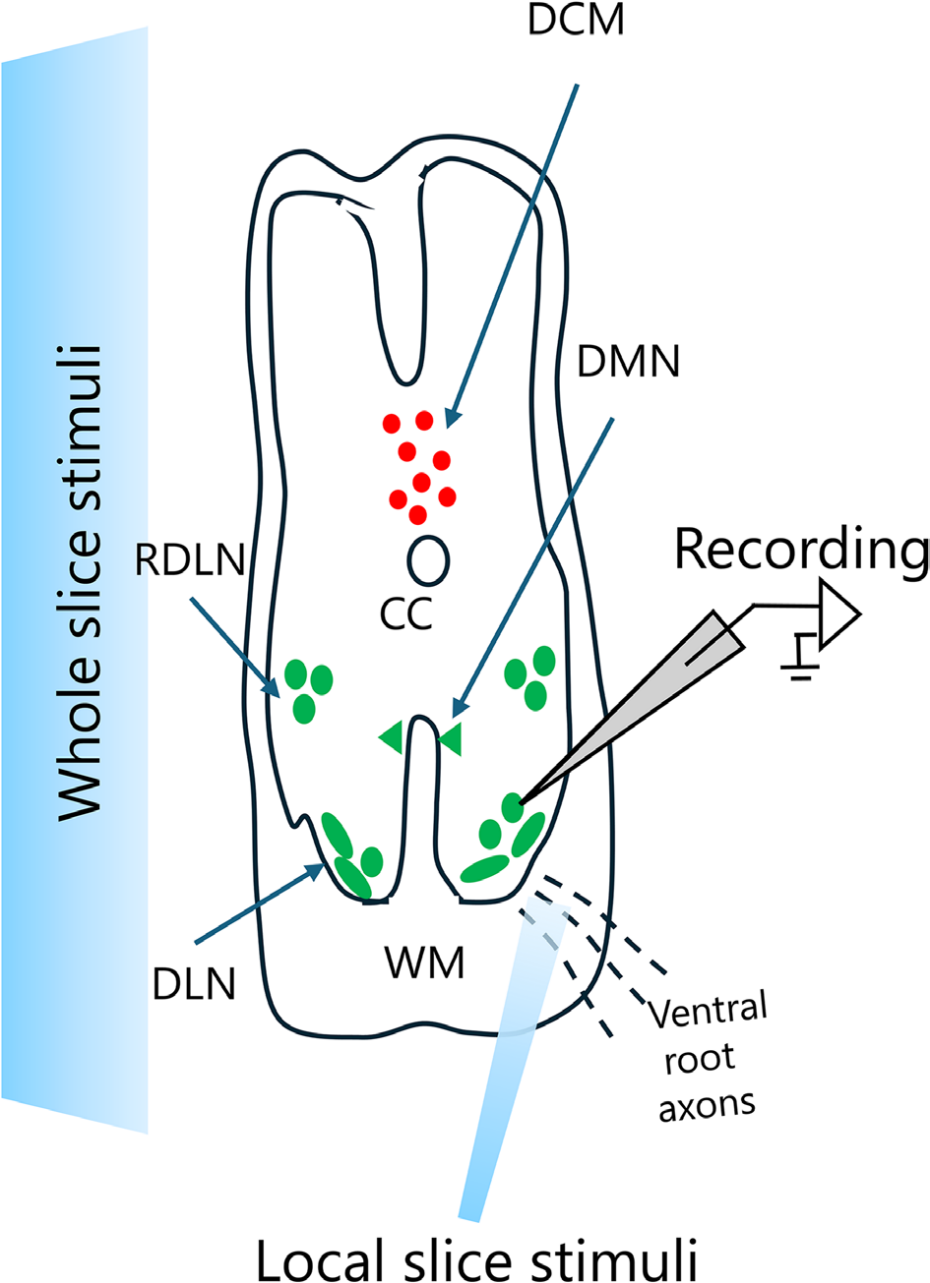
Schematic presentation of localized optogenetic stimulation targeting DLN motor neurons. The spinal cord slice is shown with major anatomical landmarks. Local slice stimuli (100 μm optical fiber) was positioned obliquely above the dorsolateral spinal region to stimulate inhibitory inputs onto cholinergic DLN motor neurons containig external urethral sphincter motor neurons (EUS–MNs). Despite targeting this area, localized 470 nm light stimulation failed to evoke IPSPs, likely due to oblique light entry, insufficient penetration, and/or limited spatial recruitment. CC, centra canal; DCM, dorsal commissure; DLN, dorsolateral nucleus; DMN, dorsomedial nucleus; RDLN, retro‐dorsolateral nucleus; WH, white matter.

ChR2 was activated using 470 nm light from an LED (M470F3, Thorlabs, Newton, NJ, USA). A schematic overview of the experimental setup for localized optogenetic stimulation is shown in Figure 2. For global stimulation, full–field illumination was triggered manually by opening the field diaphragm shutter of the microscope's epifluorescence path at maximum output. Each light pulse lasted approximately 3 s.

For localized stimulation, a 100 μm optical fiber was positioned obliquely above the slice due to spatial constraints from the objective. Light entered at an angle, causing elliptical spread and reduced spatial precision. TTL‐controlled pulses (1–5 ms) were delivered via Signal5 software (CED, Cambridge, UK).

### 2.5 Pharmacology

To dissect the neurotransmitter mechanisms underlying inhibitory postsynaptic potentials (IPSPs), pharmacological agents were applied via bath perfusion with ACSF at a flow rate of 2–3 mL/min using a Watson Marlow Peristaltic Pump 101 U set to 20–25 rpm. Each drug was administered for at least 15 min prior to electrophysiological recordings to ensure adequate diffusion and receptor blockade. Drug equilibration was verified by monitoring the stability of the pharmacological effect (i.e., consistent blockade of synaptic responses across multiple trials). Recordings were initiated only after responses stabilized, typically within 15–20 min of drug application.

The following antagonists were used: Strychnine hydrochloride (1 μM; cat. no. S8753, Sigma–Aldrich), a glycine receptor antagonist; bicuculline methiodide (10 μM; cat. no. 2503, Tocris Bioscience, Bristol, UK), a GABA_A_ receptor antagonist; CNQX (10 μM; cat. no. 0190, Tocris Bioscience), an AMPA/kainate receptor antagonist; and AP5 (10 μM; cat. no. 0106, Tocris Bioscience), an NMDA receptor antagonist.

Drug concentrations were selected based on prior studies and validated by observing consistent blockade of the respective receptor–mediated responses (Inquimbert et al., 2007).

### 2.6 Data analysis

Data were analyzed offline using Signal software (version 5.0, CED, Cambridge, UK), the same platform used for acquisition, to maintain consistency in stimulus timing and waveform alignment. IPSPs were classified as “sustained” if hyperpolarization was maintained throughout the entire duration of light stimulation (3 s), or “transient” if hyperpolarization decayed significantly before stimulus cessation despite continued optogenetic activation. Due to the exploratory nature of the study, representative examples are presented without statistical evaluation.

## 3 RESULTS

### 3.1 Optogenetically evoked inhibition in EUS motor neurons

We recorded from 31 EUS–MNs in oblique L6‐S1 spinal cord slices from VGAT–ChR2 × ChAT–GFP mice (*n* = 8 mice; 5 males, 3 females) across 5 separate experimental sessions, and successfully recorded in 16 EUS‐MNs (51.6%; 16/31 neurons), with unsuccessful recordings in the other 15 neurons due to technical factors including cell damage during patch clamp approach, insufficient ChR2 expression levels, or slice preparation‐related cell viability issues. The dataset included 16 neurons that exhibited light‐evoked inhibitory postsynaptic potentials (IPSPs) in response to global VGAT‐ChR2 activation in spinal cord slices from both male (*n* = 12 neurons) and female (*n* = 4 neurons) animals.

**FIGURE 3:**
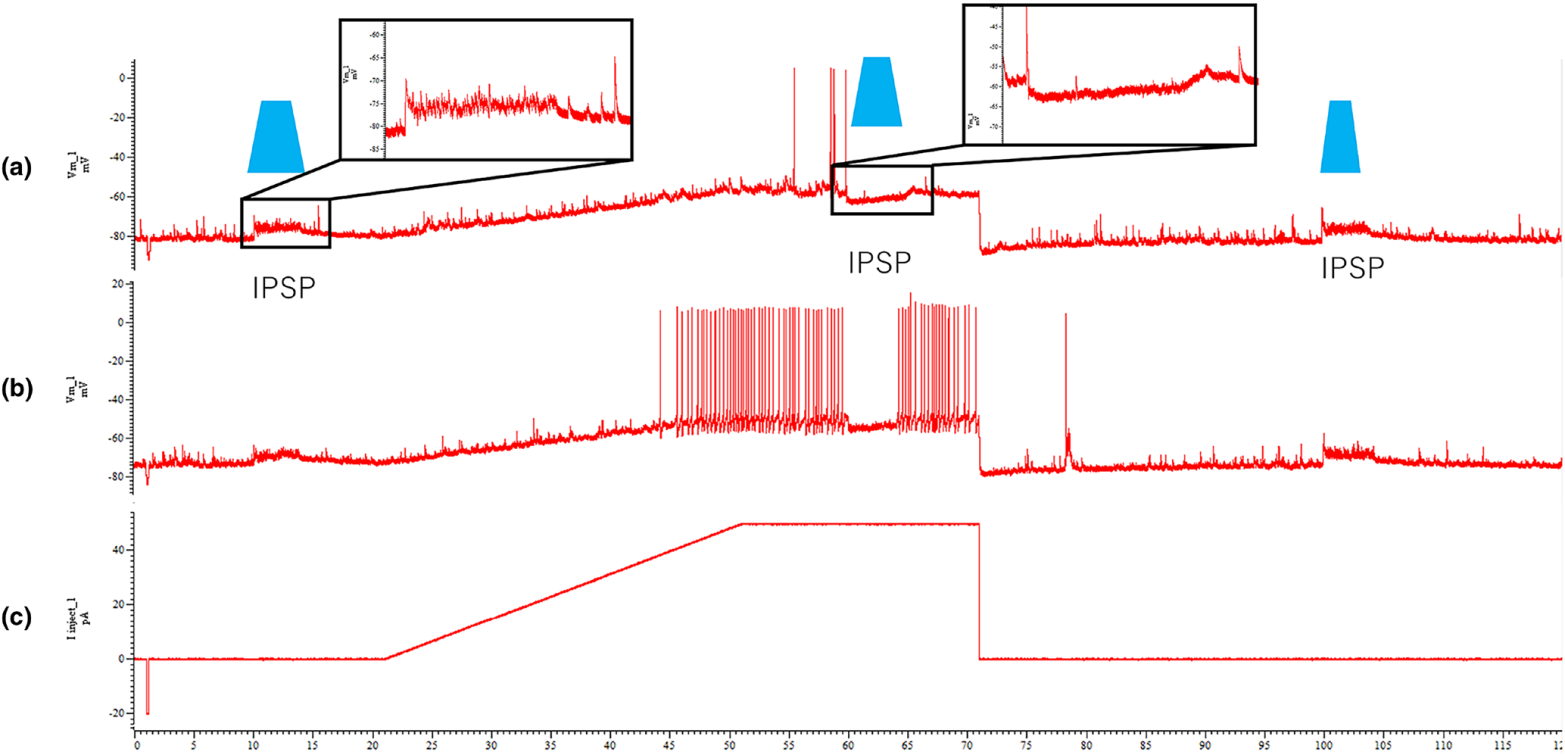
Representative recording of ChR2–evoked inhibition in an EUS motor neuron. (a) Light stimulation (470 nm, 3 s) evoked a sustained reversed IPSP when the neuron was held at subthreshold membrane potential (approximately −60 mV). Note that the IPSP appears as a depolarization due to the reversal potential being more positive than the holding potential; however, at more depolarized potentials, it manifests as hyperpolarization. Black‐outlined insets display enlarged views of the IPSP responses: the left inset shows the detailed response characteristics without current injection, and the right inset shows the response during current injection. (b) During tonic firing induced by current injection (50 pA), the same stimulation suppressed action potentials. (c) Trace of injected current used for both (a and b), consisting of a ramp and a plateau. The same current protocol was applied in both conditions to allow direct comparison of responses with and without optogenetic stimulation. Vertical axis shows membrane potential (mV) for (a and b), and injected current (pA) for (c). Horizontal axis represents time (s). Blue trapezoid labels indicate the duration of blue light illumination.

Among these 16 responsive cells, response patterns were distributed as follows: 9/16 (56.3%) exhibited sustained IPSPs, 4/16 (25.0%) showed transient IPSPs, and 3/16 (18.7%) were classified as ambiguous due to variable responses. A representative example of IPSP‐exhibiting neurons is shown in Figure 3. At subthreshold membrane potential, a 470 nm light stimulus (3 s duration) evoked a large sustained IPSP (Figure 3a). When tonic firing was induced by injecting depolarizing current (50 pA), the same light stimulus abolished spiking activity (Figure 3b), with action potential firing resuming after the stimulus was turned off (Figure 3c). These results indicate that VGAT–positive inhibitory interneurons exerted robust and reversible inhibition over EUS–MNs under both resting and active conditions. This pattern of robust sustained inhibition was consistently observed across multiple neurons in all 5 experimental sessions.

### 3.2 Strychnine–sensitive IPSPs are glycinergic and reversible

**FIGURE 4:**
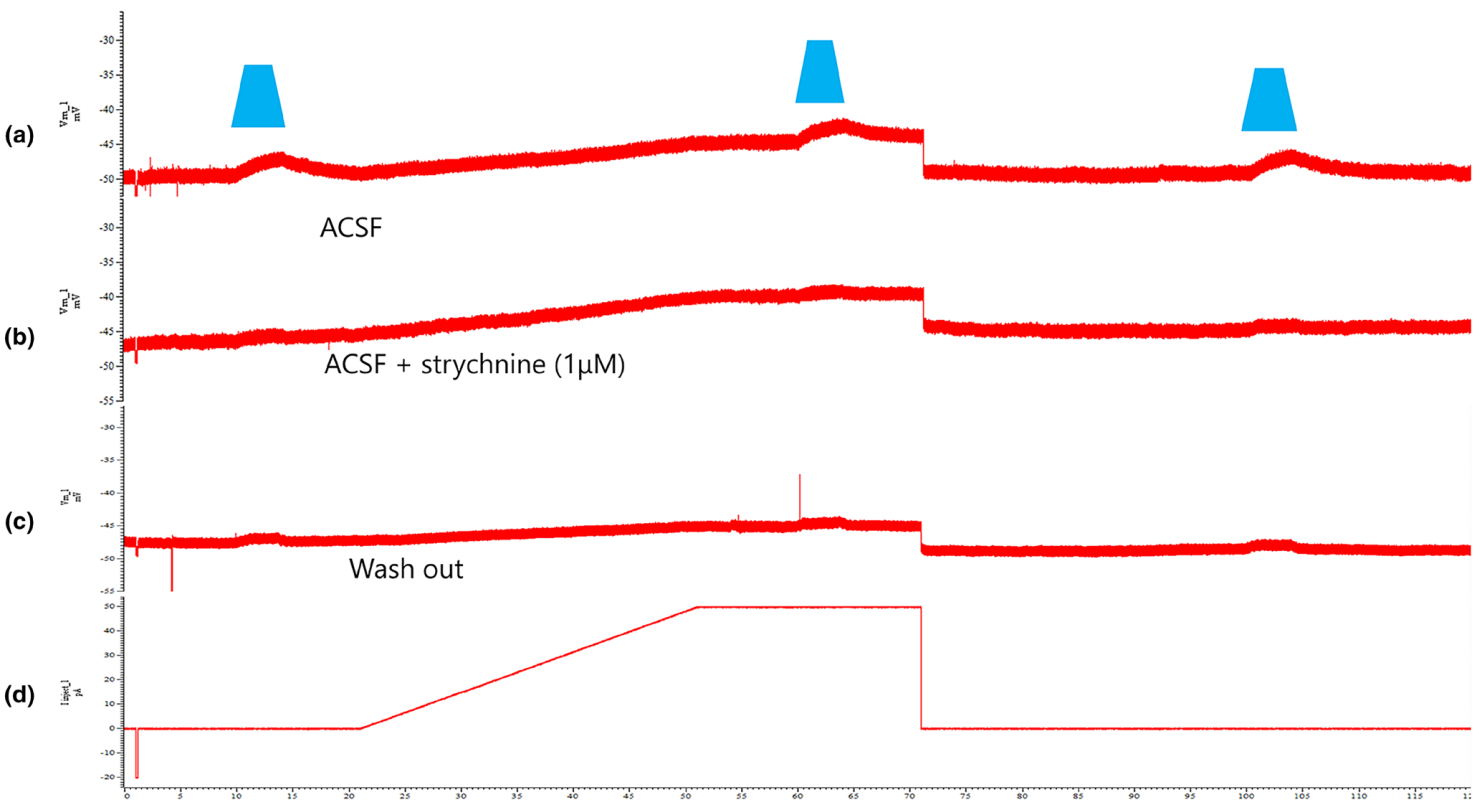
Pharmacological blockade and limited washout of glycinergic IPSPs. (a) ChR2 activation in control ACSF evoked a sustained IPSP (resting membrane potential approximately −50 mV). (b) Bath application of strychnine (1 μM) abolished the IPSP. (c) Upon washout of strychnine, the IPSP showed minimal recovery, suggesting incomplete drug clearance or receptor desensitization. (d) Trace of injected currents. Vertical axis shows membrane potential (mV) for (a–c), and injected current (pA) for (d). Horizontal axis represents time (s). Blue trapezoid labels indicate the duration of blue light illumination.

To identify the neurotransmitter basis of the IPSPs, we applied receptor–specific antagonists during recordings. In Figure 4, light–evoked IPSPs observed under control conditions were abolished by bath application of strychnine (1 μM), a selective glycine receptor antagonist. The response showed minimal recovery after washout, suggesting either incomplete drug clearance or long–lasting receptor desensitization. This finding confirms a strong glycinergic component underlying the sustained inhibitory input to EUS–MNs. This strychnine‐sensitive response was reproducibly observed in *n* = 4 neurons across 3 separate experimental sessions.

### 3.3 Dissection of inhibitory and excitatory components by multistep pharmacology

**FIGURE 5:**
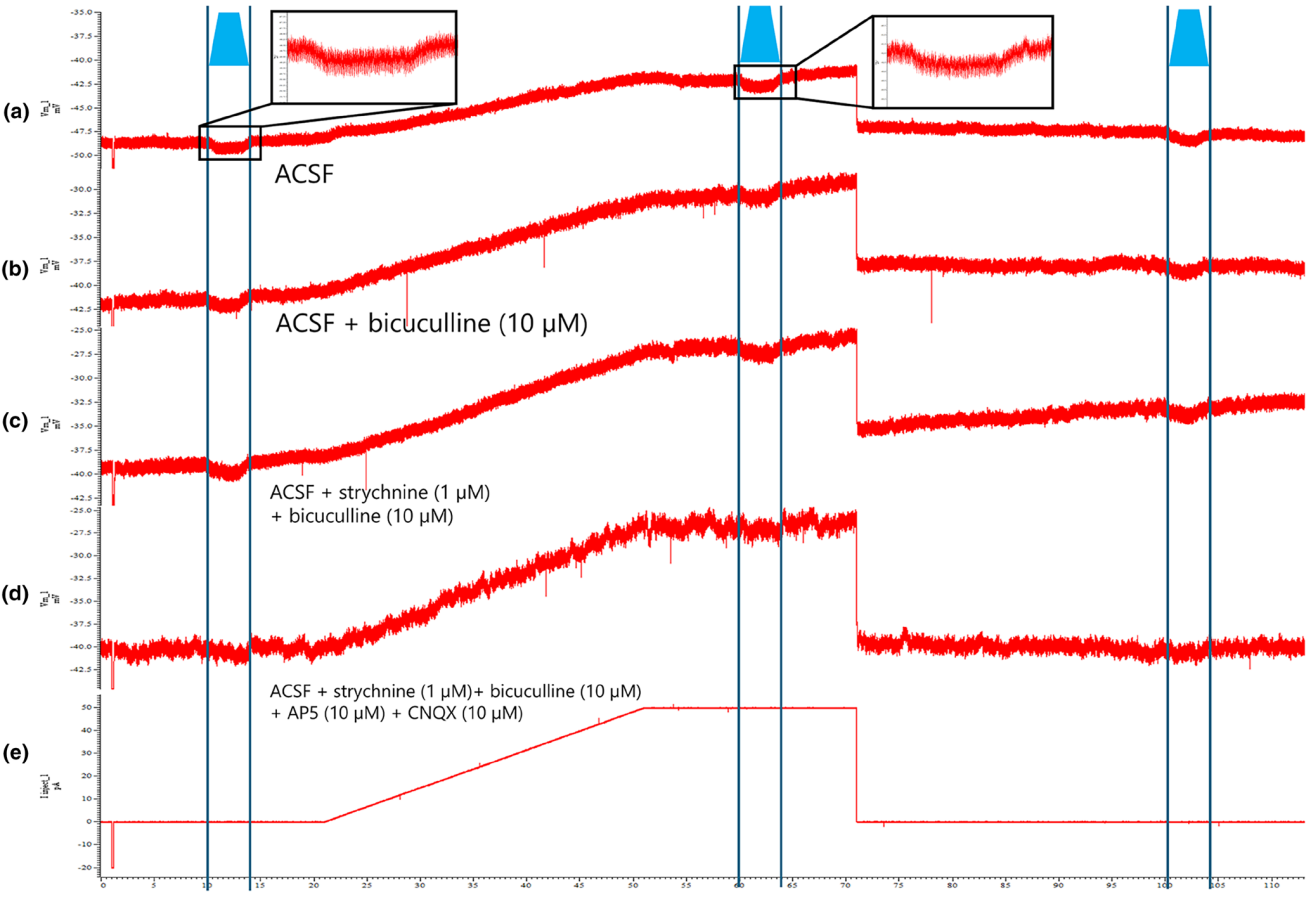
Pharmacological analyses of optogenetically evoked IPSPs. (a) Light stimulation in ACSF evokes an IPSP (resting membrane potential approximately −47 mV). Black‐outlined insets display enlarged views of the IPSP responses under different pharmacological conditions: The left inset shows the response without current injection, and the right inset shows the response during current injection. (b) Bicuculline (10 μM) reduces the IPSP amplitude, suggesting a GABA_A_ component. (c) Co‐application of bicuculline and strychnine (1 μM) greatly reduces the IPSP, indicating a mixed glycinergic and GABAergic input. (d) Addition of CNQX (10 μM) and AP5 (10 μM) shows minimal additional effects, excluding the possibility of glutamatergic involvement. (e) Trace of the injected current. Vertical axis shows membrane potential (mV) for (a–d), and injected current (pA) for (e). Horizontal axis represents time (s). Blue trapezoid labels indicate the duration of blue light illumination.

In a separate series of recordings, we systematically applied receptor antagonists to dissect the contributions of GABAergic and glutamatergic components (*n* = 5 neurons from 3 separate experimental sessions). Bath application of bicuculline (10 μM) partially reduced the IPSP amplitude (Figure 5b), and subsequent co‐application of strychnine (1 μM) abolished the IPSP entirely (Figure 5c). Further addition of the glutamatergic antagonists CNQX (10 μM) and AP5 (10 μM) had no additional effects (Figure 5d), indicating that IPSPs were not contaminated by excitatory postsynaptic potentials (EPSPs).

These findings confirm that optogenetically evoked IPSPs in EUS–MNs are mediated by a combination of GABA_A_ and glycine receptors, with no detectable glutamatergic contribution under our recording conditions.

### 3.4 Differential pharmacological sensitivity between sustained and transient responses

**FIGURE 6:**
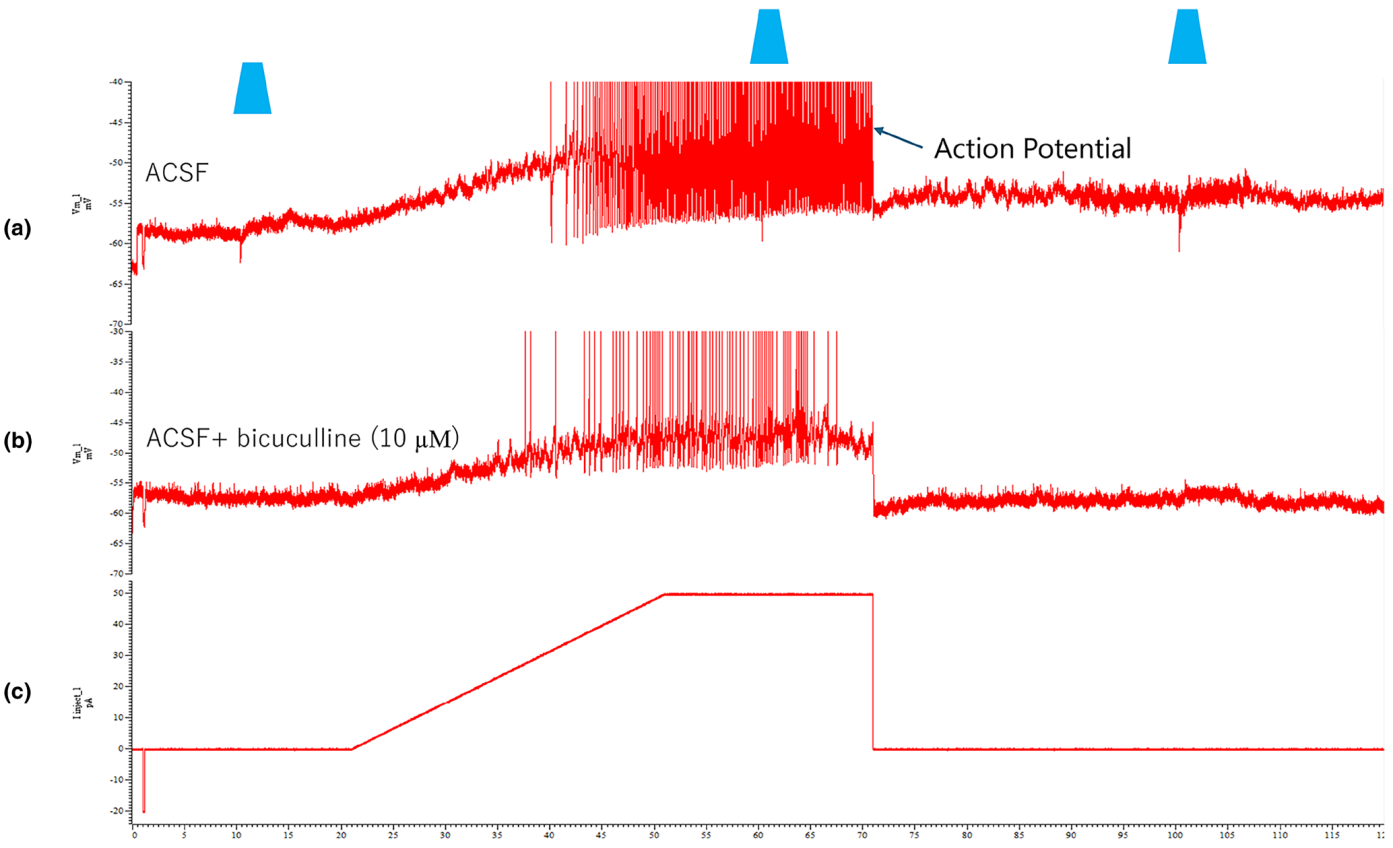
Selective suppression of transient IPSPs by bicuculline. (a) Whole–slice light stimulation in ACSF evoked a transient hyperpolarizing response (resting membrane potential approximately −40 mV) followed by tonic firing in an EUS motor neuron. (b) After bath application of bicuculline (10 μM), the transient hyperpolarizing response was abolished, while tonic firing continued with a lower frequency. (c) Overlay of the responses from control ACSF and bicuculline conditions, aligned to light stimulus onset, demonstrates selective elimination of the transient inhibitory component by GABA_A_ receptor blockade while preserving the excitatory phase, supporting the conclusion that transient IPSPs in this neuron are primarily mediated by GABAergic transmission. Blue trapezoid labels indicate the duration of blue light illumination.

Neurons exhibiting transient IPSPs showed a distinct pharmacological profile compared to those with sustained inhibition. In a representative example (Figure 6), whole–slice light stimulation evoked a fast, transient IPSP that was eliminated by bicuculline (10 μM), indicating that the response was primarily mediated by GABA_A_ receptors. Tonic firing continued after bicuculline application but at a reduced frequency (Figure 6a,b). Overlaying the initial responses from control and drug conditions revealed selective blockade of the transient hyperpolarizing component (Figure 6c). This GABA_A_ receptor‐mediated transient response pattern was observed in 4/16 neurons (25.0%) across 3 separate experimental sessions.

These observations suggest a functional segregation of inhibitory mechanisms in EUS–MNs: that is, transient IPSPs are largely GABAergic, while sustained inhibition often involves both GABAergic and glycinergic components, with glycinergic signaling playing a prominent role in maintaining prolonged hyperpolarization.

## 4 DISCUSSION

In this study, we characterized inhibitory synaptic inputs to cholinergic motor neurons in the DLN of the L6‐S1 spinal cord using a combination of optogenetic stimulation and patch‐clamp recording techniques in oblique spinal cord slices. We previously reported that the spinal DLN, which corresponds to a portion of Onuf's nucleus in humans, contains motor neurons innervating the EUS in mice using retrograde viral tracing techniques (Miyazato et al., 2009; Schellino et al., 2020). Therefore, we recorded neuronal activity of cholinergic motor neurons in the DLN region of L6‐S1 spinal cord slices in this study. Our findings provide novel insights into the pharmacological properties of inhibitory inputs to EUS‐MNs, revealing distinct glycinergic and GABAergic components. Here, we discuss the implications of our results for understanding inhibitory control mechanisms in the spinal cord circuitry governing the EUS function, while addressing technical considerations and limitations of our methodological approach.

### 4.1 Pharmacological dissection of inhibitory inputs to DLN motor neurons

Our optogenetic activation of inhibitory inputs to EUS‐MNs generated robust IPSPs that were differentially sensitive to glycinergic or GABAergic antagonists. The observation that strychnine application partially reduced IPSP amplitude, while subsequent bicuculline administration completely abolished the remaining response, indicates the presence of both glycinergic and GABAergic components in these inhibitory inputs. This mixed neurotransmitter profile aligns with previous studies demonstrating that glycine and GABA often co‐localize at inhibitory synapses in the spinal cord (González‐Forero & Alvarez, 2005; Muller et al., 2008).

The temporal characteristics of observed IPSPs‐specifically the presence of both transient and sustained responses‐provide functional evidence for the biophysical differences between glycinergic and GABAergic signaling. The observation that transient IPSPs are predominantly GABAergic while sustained IPSPs contain both GABAergic and glycinergic components suggests complex interactions between different inhibitory pathways converging onto EUS‐MNs.

The selective sensitivity of transient IPSPs to bicuculline suggests a specialized role for GABAergic signaling in phasic inhibition of EUS‐MNs. Conversely, the mixed pharmacology of sustained IPSPs would indicate that glycinergic transmission may be crucial for maintaining prolonged hyperpolarization. This functional segregation could have significant implications for controlling EUS activity during different phases of micturition. Specifically, the fast GABAergic component might modulate rapid transitions in EUS activity. At the same time, the sustained glycinergic inhibition could facilitate prolonged EUS relaxation during voiding, as suggested by studies in other spinal motor systems (Shefchyk et al., 1998).

### 4.2 Technical considerations of combined optogenetic and electrophysiological approach

Combining VGAT‐ChR2 and ChAT‐GFP mouse models with patch‐clamp recording provided several methodological advantages for investigating inhibitory synaptic transmission. The selective activation of inhibitory interneurons using ChR2 allowed us to isolate inhibitory inputs without the confounding activation of excitatory pathways that typically occur with conventional electrical stimulation. Furthermore, the visualization of cholinergic neurons via GFP fluorescence facilitated the targeted recording from identified EUS‐MNs, enhancing the specificity of our analysis, a significant advancement over traditional bulk stimulation techniques (Montgomery et al., 2016).

A significant technical advantage of our approach was the ability to control spatiotemporal patterns of inhibitory neuron activation. By varying the duration and intensity of light stimulation, we could evoke different patterns of inhibitory responses, revealing the dynamic range of inhibitory control mechanisms. This level of precision would be challenging to achieve with traditional electrical stimulation methods, as noted by Aksoy‐Aksel in their review of optogenetic applications in synaptic circuit analysis (Aksoy‐Aksel et al., 2020).

### 4.3 Methodological limitations

Several limitations warrant considerations. First, the expression levels of ChR2 may vary across inhibitory neurons, potentially biasing our observations toward neurons with higher expression (Linders et al., 2022). Second, our whole‐cell recording approach may have altered the intracellular chloride concentration, potentially affecting the magnitude and reversal potential of inhibitory currents. This consideration is significant given the role of chloride gradients in determining the polarity of inhibitory responses (Dine et al., 2014). Future studies could address this limitation by using perforated patch‐clamp techniques to maintain endogenous chloride gradients. Third, localized optogenetic stimulation using a 100 μm optical fiber positioned obliquely above the slice failed to elicit IPSPs in EUS‐MNs (Figure 2), likely due to insufficient light penetration, angular dispersion, and/or spatial mismatch between fiber position and inhibitory terminals. This limitation necessitated reliance on full‐field stimulation in this study. Also, precise spatial control in optogenetic experiments may have been limited for characterizing inhibitory circuitry in our slice preparation due to (1) insufficient light penetration through the tissue, (2) reduced spatial recruitment of inhibitory neurons due to angular dispersion of the light path, and/or (3) spatial mismatch between inhibitory afferents and the optical fiber position. Future studies with improved optical delivery methods may allow for more precise mapping of inhibitory inputs onto EUS–MNs. Fourth, slice preparation unavoidably disrupts long‐range connections that may be important for EUS control, as recognized in similar studies (Tao et al., 2015). Also, our whole‐cell recording approach may have altered intracellular chloride concentrations, potentially affecting inhibitory current magnitude and reversal potential (Dine et al., 2014). Fifth, the current study was primarily designed for method improvement and validation rather than a comprehensive quantitative analysis of inhibitory circuits. We demonstrated the feasibility of our oblique slicing approach and characterized the basic pharmacological properties of inhibitory inputs to EUS‐MNs; thus, the methodological foundation established here warrants future studies with detailed quantitative analysis of synaptic properties and statistical comparisons. Lastly, the limited sample size and 51.6% success rate in our exploratory study might restrict the generalizability of findings. The response variability likely reflects technical challenges inherent to patch‐clamp recordings from sparsely distributed EUS‐MNs in neonatal spinal tissues. Nevertheless, despite these limitations, the combined optogenetic‐electrophysiological approach remains a powerful tool for dissecting inhibitory circuits in the spinal cord, offering advantages over traditional methods in terms of specificity and control. Thus, our results provide a foundation for future investigations into the inhibitory function of EUS‐MNs that are involved in the control of micturition.

### 4.4 Future directions

While our study focused on the pharmacological properties of inhibitory inputs rather than developmental changes, the observed heterogeneity in inhibitory transmission has essential implications for understanding developmental processes. During postnatal development, glycine receptors transition from neonatal α2‐containing to adult α1/α3‐containing forms, associated with changes in receptor kinetics and clustering (Kirsch, 2006; Schwale et al., 2016). Similarly, GABA receptors exhibit developmental changes in subunit composition that influence synaptic function (Baccei & Fitzgerald, 2004). Furthermore, the balance between glycinergic and GABAergic signaling is crucial for proper inhibitory control throughout development. This balance is regulated by chloride transporters KCC2 and NKCC1, determining whether GABA and glycine have depolarizing or hyperpolarizing effects (Delpy et al., 2008; Ferrari & Ben‐Ari, 2020). Disruptions in the developmental process or pathological conditions such as spinal cord injury in adulthood have been implicated in various neurological disorders, including those affecting micturition control (Watanabe et al., 2023). Therefore, future studies could explore the developmental trajectory of inhibitory inputs to EUS‐MNs, examining how the balance between glycinergic and GABAergic transmission changes during postnatal maturation. Additionally, investigating potential sex differences in inhibitory control mechanisms would be valuable, given the known sexual dimorphism in the DLN and the higher prevalence of urinary dysfunction in males following SCI (Hashimoto et al., 2024; Karnup et al., 2024).

In addition, in the context of urinary dysfunction following spinal cord injury (SCI), our findings may have particular relevance because SCI disrupts descending pathways that usually coordinate bladder and sphincter function, often resulting in detrusor‐sphincter dyssynergia (DSD) (Hashimoto et al., 2024; Hsiang & Vizzard, 2024). Understanding the pharmacological properties of inhibitory inputs to EUS‐MNs could inform therapeutic strategies to restore proper EUS relaxation during voiding. For instance, agents that selectively enhance glycinergic transmission might facilitate sustained EUS relaxation, improving voiding efficiency in patients with DSD, as suggested by studies exploring pharmacological interventions for bladder dysfunction (Miyazato et al., 2009).

Another promising direction would be to combine our approach with anatomical tracing techniques to identify the specific sources of glycinergic and GABAergic inputs to EUS‐MNs. This could reveal whether these inputs arise from distinct interneuron populations or whether single interneurons co‐release both transmitters, as observed in other spinal circuits (Simonnet et al., 2020). Such information would provide a complete understanding of the inhibitory circuitry controlling EUS function.

In conclusion, our study demonstrates the utility of combining optogenetic stimulation with patch‐clamp recording to investigate inhibitory synaptic transmission in spinal cord slice preparations. By revealing the distinct pharmacological properties of inhibitory inputs to EUS‐MNs, our findings contribute to the understanding of neural control mechanisms for LUT function and may inform future therapeutic strategies for urinary dysfunction.

## CONFLICT OF INTEREST STATEMENT

The authors declare no conflicts of interest.

## Data Availability

The data that support the findings of this study are available from the corresponding author upon reasonable request. Representative electrophysiological recordings demonstrating the methodological approach are presented in Figures 3, 4, 5, 6.
